# Warmer and Wetter Soil Stimulates Assimilation More than Respiration in Rainfed Agricultural Ecosystem on the China Loess Plateau: The Role of Partial Plastic Film Mulching Tillage

**DOI:** 10.1371/journal.pone.0136578

**Published:** 2015-08-25

**Authors:** Daozhi Gong, Weiping Hao, Xurong Mei, Xiang Gao, Qi Liu, Kelly Caylor

**Affiliations:** 1 State Engineering Laboratory of Efficient Water Use of Crops and Disaster Loss Mitigation/MOA Key Laboratory for Dryland Agriculture, Institute of Environment and Sustainable Development in Agriculture, Chinese Academy of Agriculture Science, Beijing, 100081, P.R. China; 2 Department of Civil and Environmental Engineering, Princeton University, Princeton, 08544, United States of America; Tennessee State University, UNITED STATES

## Abstract

Effects of agricultural practices on ecosystem carbon storage have acquired widespread concern due to its alleviation of rising atmospheric CO_2_ concentrations. Recently, combining of furrow-ridge with plastic film mulching in spring maize ecosystem was widely applied to boost crop water productivity in the semiarid regions of China. However, there is still limited information about the potentials for increased ecosystem carbon storage of this tillage method. The objective of this study was to quantify and contrast net carbon dioxide exchange, biomass accumulation and carbon budgets of maize (*Zea maize* L.) fields under the traditional non-mulching with flat tillage (CK) and partial plastic film mulching with furrow-ridge tillage (MFR) on the China Loess Plateau. Half-hourly net ecosystem CO_2_ exchange (NEE) of both treatments were synchronously measured with two eddy covariance systems during the growing seasons of 2011 through 2013. At same time green leaf area index (GLAI) and biomass were also measured biweekly. Compared with CK, the warmer and wetter (+1.3°C and +4.3%) top soil at MFR accelerated the rates of biomass accumulation, promoted greater green leaf area and thus shortened the growing seasons by an average value of 10.4 days for three years. MFR stimulated assimilation more than respiration during whole growing season, resulting in a higher carbon sequestration in terms of NEE of -79 gC/m^2^ than CK. However, after considering carbon in harvested grain (or aboveground biomass), there is a slight higher carbon sink (or a stronger carbon source) in MFR due to its greater difference of aboveground biomass than that of grain between both treatments. These results demonstrate that partial plastic film mulched furrow-ridge tillage with aboveground biomass exclusive of grain returned to the soil is an effective way to enhance simultaneously carbon sequestration and grain yield of maize in the semiarid regions.

## Introduction

There are increasingly considerable concerns on agricultural practices to improve the carbon storage of cultivated soils as it is one of the greatest potential methods to sequester carbon in cropland [1–3]. Recently several studies on agronomic practices such as conservation tillage and fertilizer [4], crop covering and rotation [5], manure and crop residue applications[6], and irrigation [7–8] have been qualified the reductions in greenhouse gas emission and/or the increases of removed carbon from atmosphere. However, there is still a lack of information about the potential carbon sequestration, resulting from the transition from traditional non-mulching with flat tillage to partial plastic film mulching with furrow-ridge tillage in the semiarid regions of China [9–10].

To meet the increasing food demand while solving water crises, a newly agricultural practice, combining partial plastic film mulching with furrow-ridge tillage (MFR), has been developed to effectively collect rainwater, decrease unproductive soil evaporation and boost crop production [11–15]. It has almost replaced the traditional cultivation involving non-mulching with flat tillage which was the main agriculture practice used in semiarid regions of China. The area of MFR reached over 2,000,000 ha in 2014. This kind of tillage alters water and heat processes at the interface between soil and atmosphere which results in increments of abilities to conserve carbon, water and energy, promote crop development, and significantly affects the carbon balance of cultivated land in the end. Quantifying the effects of MFR on carbon budget of agricultural ecosystem is essential for evaluating its atmosphere environmental effects and developing carbon management strategies in semiarid regions of China.

At present, there are few studies on comparing the carbon fluxes and balance between the traditional and the innovative tillage in agro-ecosystems at one site with two eddy covariance systems. In this study, a fully based-sensors approach was used to investigate short-term effects (3 years) of maize (*Zea maize* L) change from conventional non-mulching with flat tillage to MFR on carbon exchange and balance in semiarid regions of China. This study aims to quantify and contrast the amount of C sequestered by net exchange of ecosystem and biomass and the C lost through ecosystem respiration of maize fields in non-mulching with flat tillage and MFR, and to estimate the net gains/losses of carbon due to the conversion of non-mulching with flat tillage to MFR in rainfed maize fields.

## Materials and Methods

### Description of the Experimental Site and Treatments

The experiment was conducted in a rainfed spring maize field at Experimental Station of Dryland Agriculture and Environment (ESDAE), Ministry of Agriculture, P. R. China, which is located in Shouyang, Shanxi Province, North of China (37°45′58″N, 113°12′9″E, 1202 m Alt.). The experimental period covered three growing seasons: from May 1 to September 28, 2011, from May 3 to September 22, 2012 and from April 28 to September 25, 2013. The climate at the experimental station is a typical continental temperate with an average daily temperature of 7.4°C, an average annual rainfall of 481 mm and an average number of frost free days 140 d. The growing season of spring maize is usually wet with average rainfall of 330 mm and prominent southwest wind. The soil at the station is classified as a cinnamon soil with light clay loam texture and an average bulk density of 1.34 g/cm^3^. At the effective rootzone depth (0–100 cm), the average volumetric soil water content at field capacity and wilting point were 36.0% and 12.0%, respectively. As to plough soil layer (0-30cm), initial soil pH, soil organic C and N were 8.3, 5.22g/kg and 0.84g/kg, respectively.

The experiment design was consisted of two treatments: planting in non-mulching with flat tillage (CK), and planting in partial plastic film mulching with furrow-ridge tillage (MFR). The type of experimental design used in this study is the simply contrast method for one factor (conventional and newly tillages). In CK treatment, maize was sowed in north-south rows with a distance between ones equal to 50 cm and a space between two plants within rows of 30 cm. As to MFR treatment, maize was sowed in both sides of each plastic film mulched ridge. The maize sowing rate was 66667seeds/ha for both treatments and three years. The widths of ridges and furrows are about 60 cm and 40 cm, respectively, and the height of ridge is about 8–12 cm. Only the ridge is covered by plastic film in MFR. According to observations for CO_2_/H_2_O fluxes at thousands of sites in the world, each treatment had one plot without replications due to the limited number of expensive eddy covariance systems and large area for meeting fetch standard of their installation. Although there was no duplication for each treatment, long term observation series of eddy covariance system should compensate for this shortcoming. The area of each experiment plots is about 3.0 ha, of which length and width is 200 m and 150 m respectively. It thus meets the minimum fetch requirement of eddy covariance system installation. The precipitations were 496 mm, 417 mm and 516 mm, respectively, for the three growing seasons from 2011 to 2013. Due to higher top soil temperature and moisture than CK, MFR enhanced the growth rate and thus shortened the growing seasons of maize by an average value of 10.4 days for three years (Table 1).

**Table 1:** The dates of maize sowing and physiological mature and soil water content and temperature for both treatments (CK and MFR) from 2011 to 2013.

Year	Treatments	Sowing date	Physiological maturity date	Total days of growing season	Volumetric soil water content (0-10cm, %)	Soil temperature (0-10cm, °C)
2011	CK	May 1	September 29	151 a	19.3 b	18.8 b
	MFR	May 1	September 18	140 b	22.6 a	20.0 a
2012	CK	May 3	September 22	142 a	21.5 b	19.3 b
	MFR	May 3	September 10	131 b	26.1 a	20.6 a
2013	CK	April 28	September 25	150 a	22.1 b	19.6 b
	MFR	April 28	September 15	140 b	27.3 a	21.0a
Average	CK			147.7a	21.0 b	19.2 b
	MFR			137.3b	25.3 a	20.5 a

Notes: CK and MFR stand for planting in non-mulching with flat tillage and plastic film partial mulching with furrow-ridge tillage, respectively. Figures are mean values, n = 5 for total days of growing season and n = 3 for soil water content and temperature. Letter “a” and “b” stand for significant levels.

### Environmental Variables Measurements

Half-hourly meteorological variables were obtained by an automatic weather station (Campbell Scientific Inc., Logan, UT, USA) nearby the experimental plots. Solar radiation (R_s_) was measured with a Silicon Pyranometer (LI200X, LI-COR, Inc., Lincoln, NE, USA) and precipitation (P) was registered with a pluviometer (RGB1, Campbell Scientific Inc., Logan, UT, USA). Air temperature (T_a_) and relative humidity (RH) were measured using a Vaisala probe (HMP45C, Vaisala Inc., Tucson, AZ, USA). Wind speed (u) and its direction (w) were measured using a cup anemometer and a wind vane (03002-L, R. M. Young Inc., Traverse, MI, USA), respectively. Photosynthetically active radiation (PAR) also was measured with a quantum senor (LI 190SA, LI-COR, Inc., Lincoln, NE, USA). All variables were monitored at 2 m above the surface of grassland and recorded in a data-logger (CR10RX, Campbell Scientific Inc., Logan, UT, USA).

#### Measurements of net ecosystem exchange of carbon dioxide (NEE)

In the central of each plots, fluxes of carbon dioxide, vapor and heat were measured by an open-path eddy covariance system (Campbell Scientific Inc., Logan, UT, USA) mounted on a tower, which consist of a CO_2_/H_2_O infrared analyzer (Li-7500, LI-COR, Inc., Lincoln, NE, USA) and a three-dimensional sonic anemometer (CSAT-3, Campbell Scientific Inc., Logan, UT, USA). The sensor height was adjusted to keep the relative height of 0.5 m between sensors and maize canopy constant at interval of one or two weeks. Specific time length depended on the increments of canopy height. The observation site had a wide fetch of at least 75 m in all directions, which allowed us to neglect advection in the maize field. Top soil temperatures (2cm and 6cm below soil surface) were measured with temperature probes (TCAV, Campbell Scientific Inc., Logan, UT, USA) at three different points; the averaged data were used in our analysis. Half-hourly volumetric water contents (*θ*) at three points in each plot were monitored at a depth of 5cm in the maize field through soil water sensors (ML2X Theta probe, Delta-T Devices Ltd, London, UK). The measurements were calibrated by oven drying method. Before the experiment, two sets of eddy covariance systems were calibrated to acquire precise data at the same standard and decrease the errors depend on different instruments. Calibration of the two sets of gas analyzers was performed using chemical absorption columns for zero values and a compressed gas source of CO_2_ at 600±10 mmol/mol (Linde, Linde Gas Group, England) and a portable dew point generator (Li-610, LI-COR, Inc., Lincoln, NE, USA). Fluctuations in wind speed, sonic virtual temperature, and CO_2_/ H_2_O concentrations were sampled with the digital micro-logger at 10 Hz.

Flux values were recorded at 30 min intervals with a data-logger (CR5000, Campbell Scientific Inc., USA). Firstly, WPL density and advective corrections were applied to fluxes of CO_2_ [16–18]. To reduce errors related to insufficient turbulent mixing at night, according to analysis of Barford et al. (2003) and Verma et al. (2005), a threshold mean wind speed (U) of 2.5 m/s (corresponding to a friction velocity, u* of 0.25 m/s, approximately) was also selected. While U less than 2.5 m/s, CO_2_ flux data were deleted [19–20]. Linear interpolations between values adjacent to missing or abnormal value(s) and mean diurnal variation (MDV) of previous or afterwards periods [21–22] were used for filling small (2–3 half-hourly means missing) and greater gaps above two hours, respectively. The net ecosystem production (NEP, positive values) is equal but opposite in sign to the net ecosystem CO_2_ exchange (NEE, negative values). Daytime estimates of ecosystem respiration (R_e_, positive values) were obtained from air temperature or soil temperature data with relationships between the night CO_2_ exchange and top soil/air temperature [23]. The gross primary productivity (GPP) was then obtained by summing R_e_ and NEP. The canopy photosynthetic assimilation is opposite to GPP.

### Green Leaf Area Index and Biomass Measurements

In the central of each plot, five maize plants were randomly selected to manually measure lengths and widths of green leaves at interval of one or two weeks during the growing season. Specific interval depended on maize growth stages and leaf growth rates. Green leaf area index (GLAI) was calculated by summing lamina length × maximum width of each leaf multiplied by an empirical factor of 0.74, and then divided by area per a plant (Eq 1) [24].

$$GLAI=0.74\times (\sum_{i=1}^{n}L_{i}\times W_{i})/(D_{row}\times S_{plant})$$ (1)

GLAI stands for green leaf area index (m^2^/m^2^); L_i_ and W_i_ stand for the length and width of the i th green leaf, respectively; D_row_ and S_plant_ stand for the distance between the two rows and the space between the plants in the row, respectively.

Total aboveground and belowground biomasses were determined from destructive samples at five sites at about two weeks intervals until harvest. A plot of 30 cm × 50 cm with a normal maize plant growing in the center was selected for each site. One shoot was cut near the soil surface and collected for each site, and then were dried at 80°C for 48 h to determine the dry matter weight. The roots were also collected at soil layer of 0–100 cm. Root dry weight was determined after drying in an oven at 80°C for 48 h. While grain was matured, two plots were harvested respectively by harvesting machine to measure total yields for each treatment. At same time, the above biomass exclusive of grain was sliced and returned to the field for the sake of biomass carbon stored in the top soil.

### Statistical Analysis

Data from randomly sampling measurements for soil and plant in each plot were subjected to analysis of variance using SAS v. 8.0 software (SAS Institute, Cary, NC, USA) and mean values were compared by least significant difference (LSD) at the 5% level. Variation of GLAI with days after sowing was also fitted with a log normal function (GLAI = a exp(-0.5(ln(DAS/DAS_0_)/b)^2^)) with three parameters including a, b and DAS_0_, which stand for maximum GLAI, increment coefficient and the days after sowing while the GLAI arrives the maximum value, respectively. Pearson correlations were used to analyze the relationships among NEE, GLAI, PAR, soil environmental factor under CK and MFR tillage to deeply explore the contribution of soil moisture and temperature to the NEE under the CK and MFR farming system. The relationship between biomass carbon and canopy assimilated carbon was also regressed with the same method.

## Results

### Meteorological Variables Changes

Table 2 showed the monthly changes of five key meteorological variables during three growing seasons of 2011–2013. Meteorological variables included solar radiation (R_s_), air temperature (T_a_) and vapor pressure deficit (VPD), photosynthetically active radiation (PAR) and rainfall. There were similar seasonal trends of all variables during three growing seasons of 2011–2013. Monthly average air temperature followed the similar trends among three growing seasons of 2011–2013, progressively increased from May to June, stabilized fluctuation in July through August and decreased to September. Comparing three growing season, average monthly temperature revealed a warmer initial period in 2012 and 2013 than 2011, a equitable hot middle period in three years and a colder ending stage in 2011 and 2012 than in 2013. Average vapor pressure deficit (VPD) decreased from May to September during three growing seasons with higher average values in 2013 than in 2011 and 2012. Solar radiation and PAR shared similar change pattern in three growing seasons, which increased rapidly from May to June and then decreased gradually to September. However, average monthly solar radiation and PAR had higher value in 2011 than in both of 2012 and 2013. Total rainfall of 2011 was slightly lower than that of 2013 and greatly higher than that of 2012, and the distributions of monthly rainfall in 2011 were more even than that in 2012 and 2013. However, rainfalls in July of 2011were greatly lower than that recorded in2012 and 2013, resulting in a slight drought for maize growth.

**Table 2:** Monthly average/total meteorological variables such as air temperature (T_a_), vapor pressure deficit (VPD), shortwave incoming radiation (R_s_), PAR and rainfall at the experimental site during whole maize growing periods of 2011–2013.

Year	Month	Average T_a_ (°C)	Average VPD (kPa)	Average R_s_ (MJ m^-2^ d^-1^)	Average PAR (mol m^-2^ d^-1^)	Total rainfall (mm)
2011	May	14.64	0.92	20.48	48.46	48.1
	June	19.96	0.99	21.81	56.75	68.4
	July	20.42	0.61	20.45	46.65	149.3
	August	18.57	0.40	16.86	32.57	119.8
	September	13.02	0.36	13.21	25.40	110.5
	Total/average	17.32	0.66	18.56	41.97	496.1
2012	May	17.07	1.01	17.57	37.88	28.7
	June	19.33	1.02	17.50	37.68	61.3
	July	20.74	0.50	14.81	32.67	215.0
	August	18.77	0.41	15.43	32.19	38.5
	September	13.30	0.40	13.80	28.92	73.0
	Total/average	17.84	0.67	15.82	33.87	416.5
2013	May	16.68	0.98	19.60	33.35	29.1
	June	18.87	0.79	18.66	32.64	97.2
	July	21.04	0.58	17.47	29.47	225.1
	August	19.36	0.71	19.85	33.23	61.7
	September	15.30	0.43	15.49	24.87	101.8
	Total/average	18.25	0.70	18.21	30.71	514.9

### Variations of Daily Soil Water Content and Soil Temperature with Rainfall

**Fig 1:**
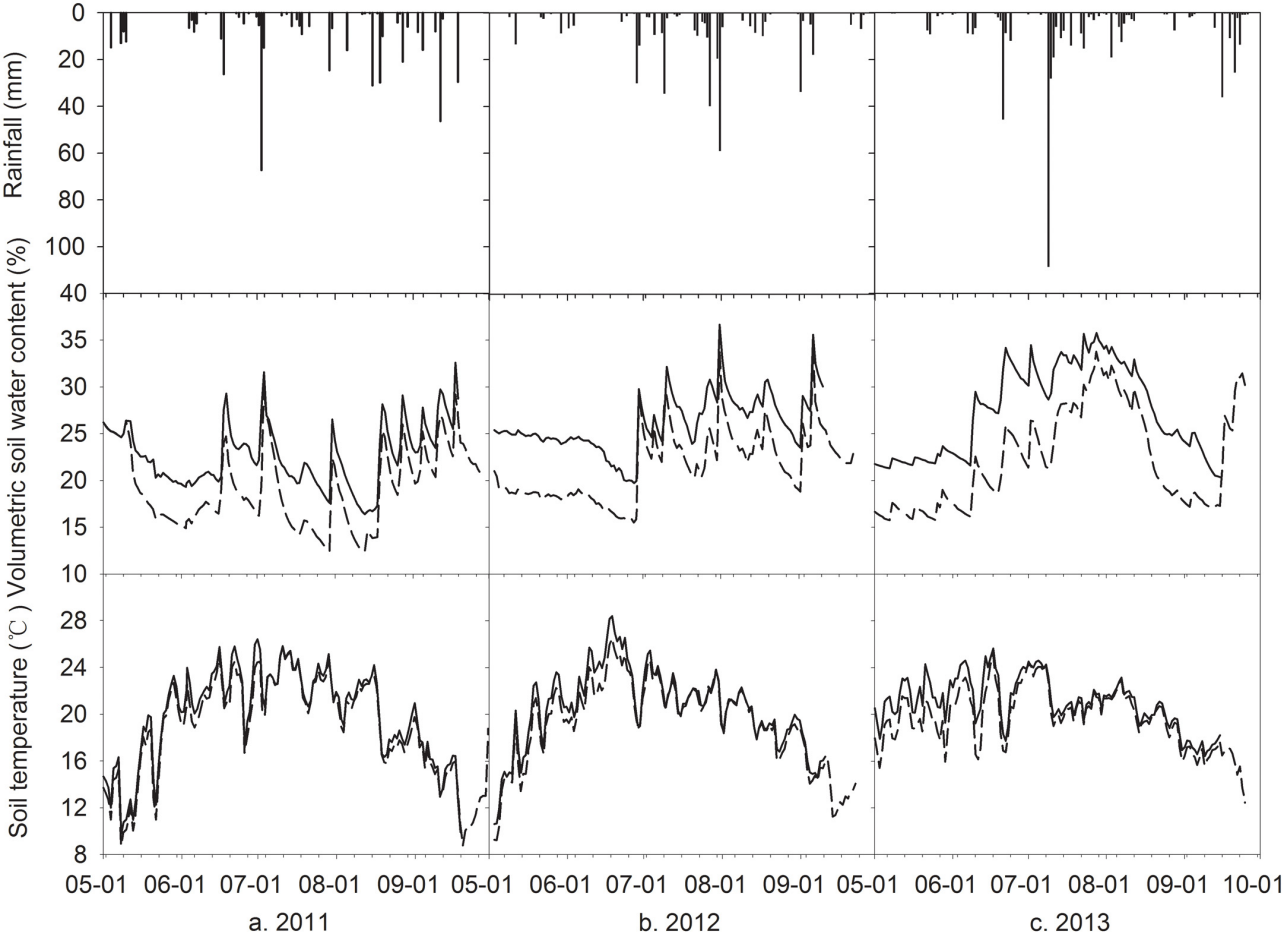
Dynamics of volumetric soil water content and temperature and rainfall during maize growing seasons of 2011–2013. Vertical bars represent rainfall, dashed and solid lines represent for CK and MFR data, respectively.

Between CK and MFR treatments, soil water content at shallow layer (0-10cm) quite differently changed during three growing seasons (Fig 1). Soil water contents in MFR always were higher than in CK except for the initial of experiment in 2011. However, both of them marked fluctuations associated with rainfalls during three growing seasons. Soil water content increased quickly to the peak values following a rainfall event, and reduced gradually till the next rainfall. The average daily soil temperature showed marked seasonal changes in growing seasons for three years and both treatments, and difference between CK and MFR had higher average value in 2011 than in 2012 and 2013. Soil temperature in the MFR was significantly higher than that in the CK during early growing season (May through July); however, in the middle growing season (August) both of them shared similar values and during the late growing season MFR had higher values than CK with a smaller difference compared to the early growing season. This phenomenon is explained by that plastic film mulching mainly controlled resistance of heat flux between ecosystem and atmosphere in the initial growing season, but canopy cover dominated it in the middle and late growing season. Totally, average volumetric soil water content and temperature in the MFR were higher by 4.3% and 1.3°C, respectively, than those of the CK during three growing seasons (Table 1).

### Green Leaf Area Index and Biomass Accumulation

**Fig 2:**
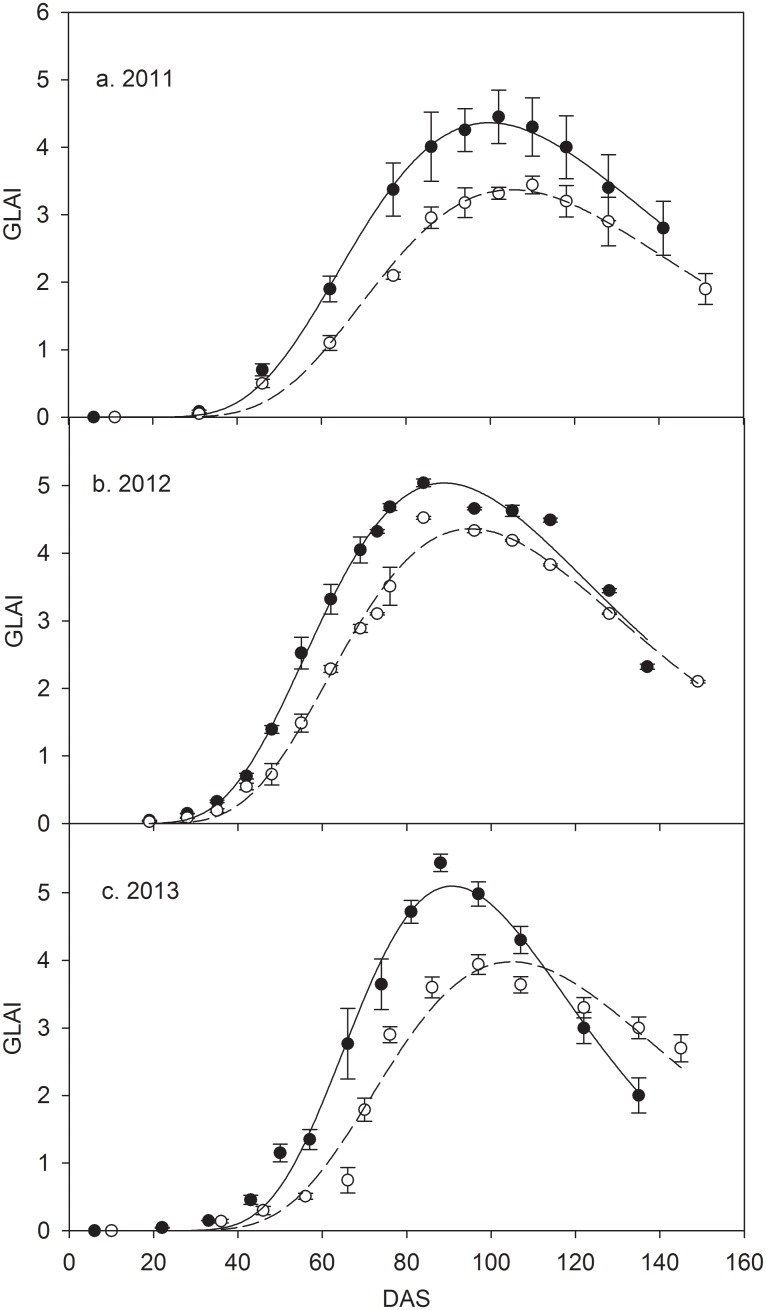
Season variations of green leaf area index (GLAI) during maize growing seasons of 2011–2013. Hollow and filled circles represent for CK and MFR data, respectively; vertical bar represents standard errors; dashed and solid lines represent the fitted curves for CK and MFR data, respectively. DAS represents days after sowing.

Green leaf area index (GLAI) for both treatments during three growing seasons are shown in Fig 2. GLAI was significantly lower in CK than MFR during the early and mid growing season but close to or higher in the late growing season due to quicker growth and shorter growing season in MFR, which resulted from its higher temperature and water status at top soil (Fig 1). There is a log normal function relationship (p<0.001) between GLAI and days after sowing (DAS) for both treatments and three growing seasons. The detailed information about the parameters and significant levels for the fitted relationships shows in the Table 3.

**Table 3:** The detailed information about the parameters and significant levels of log normal function (GLAI = aexp(-0.5(ln(DAS/DAS_0_)/b)^2^)) for the fitted relationship between GLAI and DAS.

Year	Treatments	a	b	DAS_0_	n	Significant levels
2011	CK	3.28	0.40	94.11	12	<0.0001
	MFR	4.23	0.41	93.98	12	<0.0001
2012	CK	4.33	0.43	82.31	17	<0.0001
	MFR	5.01	0.44	77.47	17	<0.0001
2013	CK	3.97	0.36	95.37	13	<0.0001
	MFR	5.09	0.31	85.50	13	<0.0001

Notes: CK and MFR stand for planting in non-mulching with flat tillage and partial plastic film mulching with furrow-ridge tillage, respectively. GLAI and DAS represent green leaf area index and days after sowing, respectively. a, b and DAS_0_ stand for maximum GLAI, increment coefficient and the days after sowing while the GLAI arrives the maximum value, respectively.

**Fig 3:**
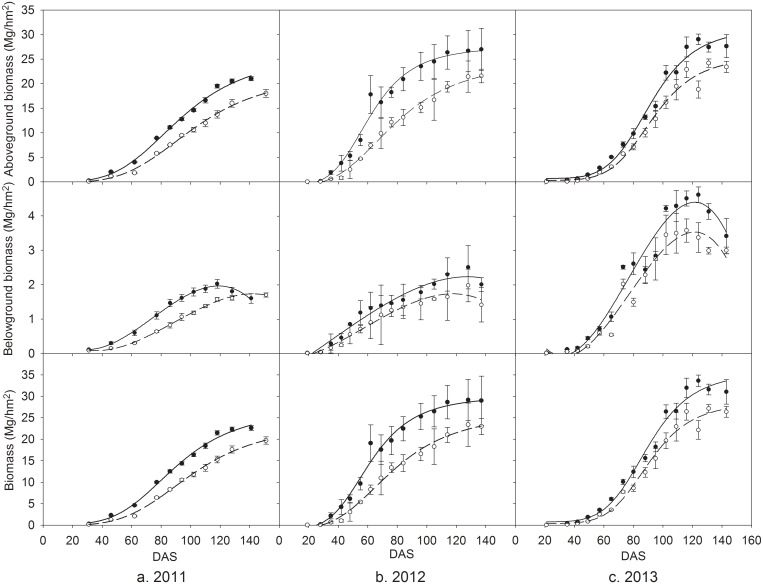
Aboveground, belowground and total biomass accumulations of spring maize during growing seasons of 2011–2013. Unfilled and filled circles represent for CK and MFR mean data, respectively; vertical bar represents standard errors; dashed and solid lines represent the fitted curves for CK and MFR data, respectively. DAS represents days after sowing.

Biomass measurements for both treatments are plotted in Fig 3. During the three whole growing seasons, aboveground and total biomass was greatly higher and belowground biomass was slightly higher at the MFR plot. However, the percentage of biomass allocated to roots was higher at the CK plot due to lower water status, which is similar to Suyker et al. (2004) results on rainfed and irrigated maize [8]. The differences in total biomass and its components between CK and MFR treatments were greatest in 2012 and smallest in 2013 because of different rainfall conditions during three growing seasons.

### Half-Hourly, Daily and Season Variations of NEE and Its Components

As shown in Table 4, daytime half-hourly NEE had better relationship with PAR and GLAI (interpolated from biweekly measurements with the fitted log normal function), but had poor relationships with soil water content and temperature. However, all relationships attained to significant level for both treatments and three growing seasons. These results suggested that NEE was affected significantly by both of environmental and biological factors. NEE is the balance between canopy photosynthesis and ecosystem respiration rates. The former is controlled mainly by GLAI and PAR, but the latter was adjusted by air/soil temperature and water content.

**Table 4:** Correlated relationships and its significant level between daytime half-hourly net ecosystem exchange of CO_2_ and environmental factors and green leaf area index (interpolated from biweekly measurements with the fitted log normal function) during whole growing seasons of 2011–2013.

Year	Treatments	Factors	R	n	Significant level
2011	CK	PAR	0.34	2850	<0.001
		GLAI	0.66		<0.001
		T_s_	0.20		<0.001
		θ_s_	0.24		<0.001
		PAR×GLAI	0.86		<0.001
	MFR	PAR	0.49	2460	<0.001
		GLAI	0.60		<0.001
		T_s_	0.35		<0.001
		θ_s_	0.26		<0.001
		PAR×GLAI	0.88		<0.001
2012	CK	PAR	0.47	2450	<0.001
		GLAI	0.62		<0.001
		T_s_	0.15		<0.001
		θ_s_	0.26		<0.001
		PAR×GLAI	0.85		<0.001
	MFR	PAR	0.57	2443	<0.001
		GLAI	0.54		<0.001
		T_s_	0.15		<0.001
		θ_s_	0.25		<0.001
		PAR×GLAI	0.85		<0.001
2013	CK	PAR	0.57	2108	<0.001
		GLAI	0.66		<0.001
		T_s_	0.11		<0.001
		θ_s_	0.19		<0.001
		PAR×GLAI	0.92		<0.001
	MFR	PAR	0.55	2189	<0.001
		GLAI	0.65		<0.001
		T_s_	0.10		<0.001
		θ_s_	0.28		<0.001
		PAR×GLAI	0.88		<0.001

Notes: CK and MFR stand for planting in non-mulching with flat tillage and partial plastic film mulching with furrow-ridge tillage, respectively. T_s_ is soil temperature (0-10cm, °C) and θ_s_ is volumetric soil water content (0-10cm, %). R is correlation coefficient and n is data number.

**Fig 4:**
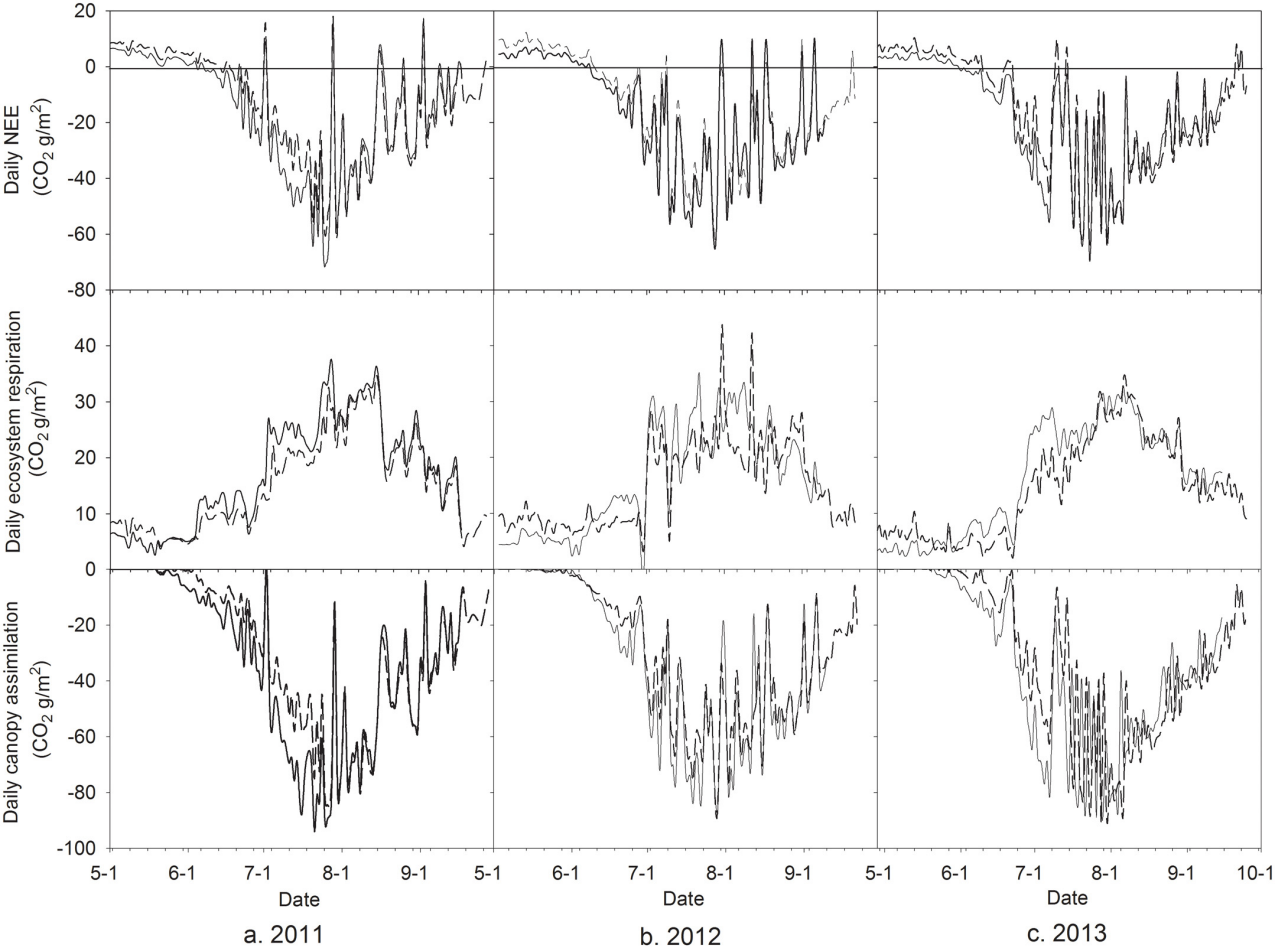
Daily net ecosystem exchange of CO_2_ and its components estimated from eddy covariance measurements during maize growing seasons of 2011–2013. Dashed and solid lines represent the curves for CK and MFR data, respectively.

Fig 4 shows the daily NEE and its components such as ecosystem respiration and canopy assimilation estimated from eddy covariance measurements. For three growing seasons, NEE and canopy assimilation of both treatments remained higher value at the initial stage, decreased quickly to the negative peak value and stabilized fluctuation over this value about two month in the middle stage and then increased gradually with leaf senescence at the late growing stage. However, ecosystem respiration changed with the reverse trend. Daily values of NEE and it components in CK treatment was higher during the early growing stage, similar or lower during middle growing season and lower during late growing season for three growing seasons.

**Fig 5:**
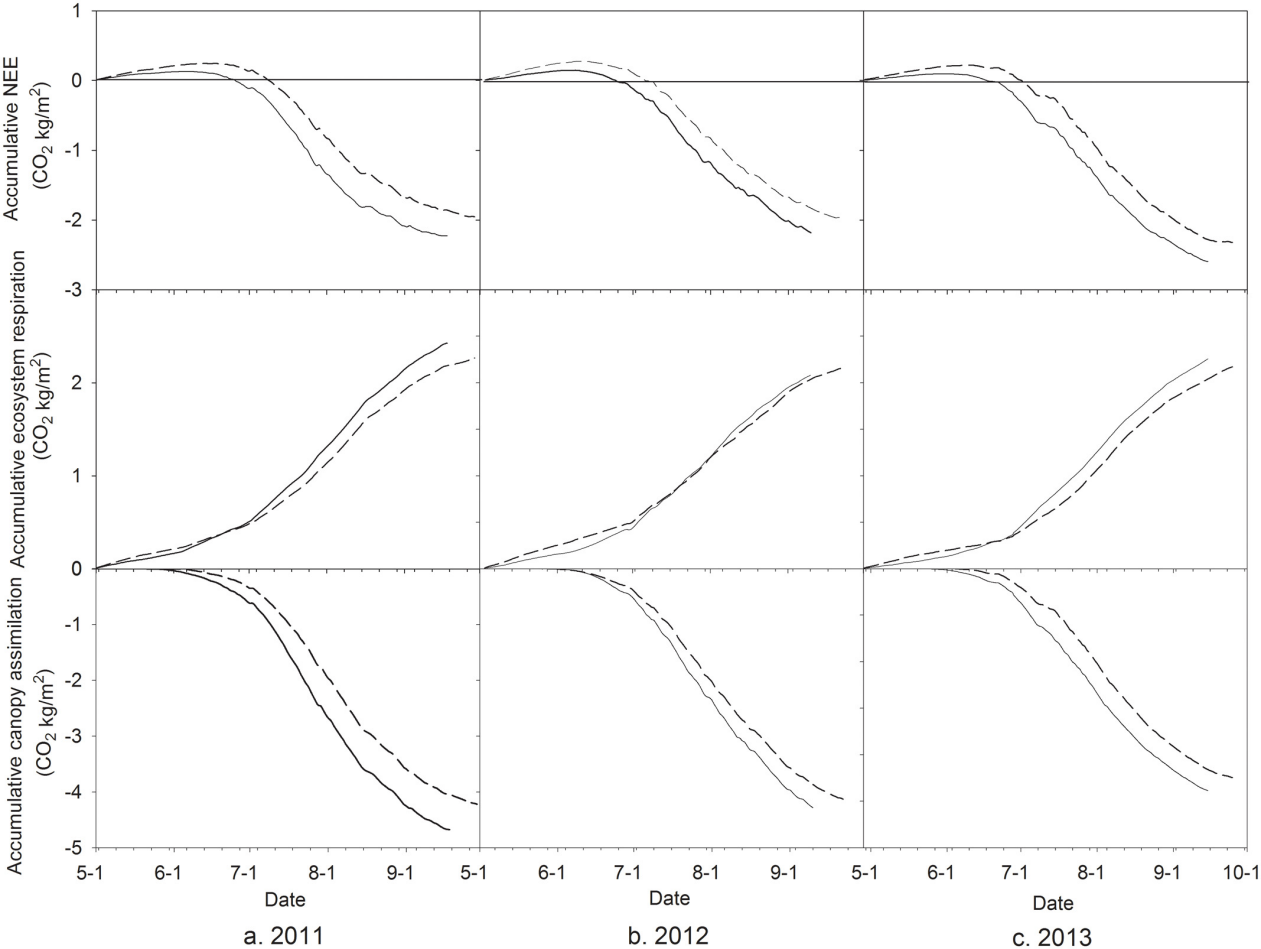
Accumulation of daily ecosystem CO_2_ flux and its components estimated from eddy covariance measurements during maize growing seasons of 2011–2013. Dashed and solid lines represent the curves for the CK and MFR data, respectively.

Accumulative values of NEE and its components are plotted in Fig 5. The maize ecosystem followed similar seasonal changes of CO_2_ uptake and release for both treatments and three years. NEE increased gradually at early growing stage, but decreased quickly during mid-growing season and slowly at late growing season. Accumulative values of daily ecosystem respiration and canopy assimilation always changed with two contrast trends. The former increased gradually but the latter decreased with days after sowing (DAS). Accumulative canopy assimilation of the MFR reached more negative value than that of the CK at the end of the growing season. However, accumulative values of daily ecosystem respiration of the MFR attained more positive value than that of the CK in the end. The difference of accumulative canopy assimilation between two treatments was greater than that of accumulative ecosystem respiration, resulting in a more negative NEE in the MFR than in the CK.

Growing stages distribution of NEE reveals that seasonal sum of carbon fixed by the ecosystem as NEE was about -2.225kgCO_2_/m^2^, -2.196 kgCO_2_/m^2^ and -2.594 kgCO_2_/m^2^, absolute vales of which was higher by 13.85%, 11.74%, and 13.08% respectively, for three years in the MFR compared to the CK (Table 5). The value NEE at all growing stages were more negative in the MFR during three growing seasons. Ecosystem respiration (R_eco_) of the MFR was always higher than that of the CK at all stages except for seedling stage during three growing seasons. This result can be explained for dominant role of plastic mulching resistance to soil CO_2_ emission at early growing season and greater plant respiration at mid and late growing seasons. Canopy photosynthesized CO_2_ and dry biomass increments at different stages were also greater in the MFR treatment for three growing seasons.

**Table 5:** Integrated ecosystem carbon flux and its components and dry biomass increments in maize fields at different growth stages of 2011–2013.

Year	Treatments	Growing stages	Period	Days	Ecosystem net CO_2_ flux (kg CO_2_ /m^2^)	Ecosystem respiration flux (kg CO_2_ /m^2^)	Canopy photosynthesis (kg CO_2_/m^2^)	Dry biomass increments (kg/m^2^)
2011	CK	Seedling	5.01–5.31	30	0.205	0.208	-0.003	0.015
		Shooting	6.01–7.03	33	-0.076	0.301	-0.376	0.165
		Heading	7.04–7.23	20	-0.611	0.390	-1.002	0.505
		Filling	7.24–9.06	45	-1.260	1.115	-2.375	0.864
		Maturity	9.06–9.29	23	-0.212	0.253	-0.465	0.091
		Total	5.01–9.29	151	-1.954	2.267	-4.221	1.640
	MFR	Seedling	5.01–5.28	28	0.117	0.145	-0.013	0.028
		Shooting	5.29–6.28	31	-0.170	0.316	-0.501	0.432
		Heading	6.29–7.16	18	-0.565	0.410	-0.994	0.538
		Filling	7.17–8.28	42	-1.370	1.173	-2.549	1.151
		Maturity	8.29–9.18	21	-0.237	0.383	-0.620	0.111
		Total	5.01–9.18	140	-2.225	2.426	-4.677	2.260
2012	CK	Seedling	5.03–5.31	29	0.239	0.247	-0.008	0.030
		Shooting	6.01–6.30	30	-0.110	0.240	-0.350	0.370
		Heading	7.01–7.19	19	-0.527	0.384	-0.911	0.937
		Filling	7.20–8.31	43	-1.284	1.000	-2.284	0.764
		Maturity	9.01–9.22	21	-0.283	0.276	-0.559	0.323
		Total	5.03–9.22	142	-1.965	2.148	-4.113	2.424
	MFR	Seedling	5.03–5.28	26	0.122	0.139	-0.010	0.030
		Shooting	5.29–6.26	29	-0.165	0.242	-0.395	0.660
		Heading	6.27–7.13	17	-0.442	0.339	-0.784	1.069
		Filling	7.14–8.23	41	-1.317	1.032	-2.365	1.104
		Maturity	8.24–9.10	18	-0.394	0.314	-0.728	0.093
		Total	5.03–9.10	131	-2.196	2.066	-4.282	2.956
2013	CK	Seedling	4.28–5.28	31	0.191	0.184	0.007	0.015
		Shooting	5.29–6.28	31	-0.122	0.177	-0.299	0.251
		Heading	6.29–7.19	22	-0.497	0.372	-0.868	0.644
		Filling	7.20–9.01	42	-1.560	1.106	-2.666	1.736
		Maturity	9.02–9.25	23	-0.306	0.320	-0.669	0.148
		Total	4.28–9.25	150	-2.294	2.158	-4.495	2.794
	MFR	Seedling	4.28–5.25	28	0.091	0.103	-0.009	0.045
		Shooting	5.26–6.24	30	-0.171	0.216	-0.371	0.310
		Heading	6.25–7.14	20	-0.589	0.468	-1.093	0.654
		Filling	7.15–8.25	41	-1.532	1.101	-2.545	1.991
		Maturity	8.26–9.15	21	-0.393	0.370	-0.755	0.260
		Total	4.28–9.15	140	-2.594	2.258	-4.773	3.260
Averages	CK	Seedling		30.0	0.212	0.213	-0.001	0.020
		Shooting		31.3	-0.103	0.239	-0.342	0.262
		Heading		20.3	-0.545	0.382	-0.927	0.695
		Filling		43.3	-1.368	1.074	-2.442	1.121
		Maturity		22.3	-0.267	0.283	-0.564	0.187
		Total		147.7	-2.071	2.191	-4.276	2.286
	MFR	Seedling		27.3	0.110	0.129	-0.011	0.034
		Shooting		30.0	-0.169	0.258	-0.422	0.467
		Heading		18.3	-0.532	0.406	-0.957	0.754
		Filling		41.3	-1.406	1.102	-2.486	1.415
		Maturity		20.0	-0.341	0.356	-0.701	0.155
		Total		137.0	-2.338	2.250	-4.577	2.825

Notes: CK and MFR stand for planting in non-mulching with flat tillage and partial plastic film mulching with furrow-ridge tillage, respectively.

### Relationship between Canopy Assimilated and Biomass Carbon at Bi-Weekly Scale

**Fig 6:**
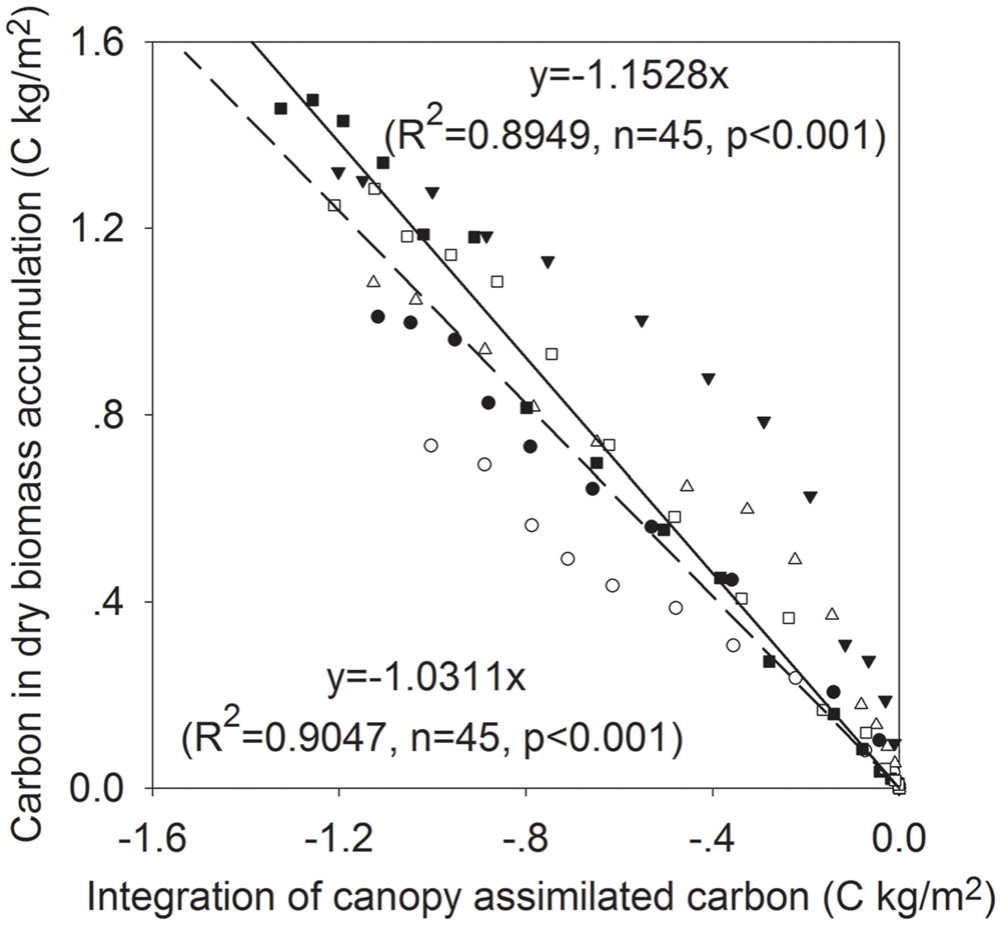
The relationships between dry biomass carbon and the sum of daily canopy assimilated carbon at different growing stages of 2011–2013. Unfilled and filled symbols represent CK and MFR data, respectively; triangle, square and circle represent 2011, 2012, and 2013, respectively. Dashed and solid lines represent the fitted curves for CK and MFR data, respectively.

The relationships between biomass carbon and canopy assimilated carbon are plotted in Fig 6. There were significantly linear relationships between biomass carbon and canopy assimilated carbon for both treatments and three years. It showed that seasonal distributions of the daily crop gain of carbon estimated from measured NEE were correlated reasonably well with the total (above and belowground) biomass. The slopes of linear regression models were -1.03 and -1.15 for CK and MFR, respectively. The slopes of linear relationships should be theoretically less than 1.0 due to plant respiration, but in this study they were slighter greater than 1.0 because of underestimation of NEE from eddy covariance measurements. The result is consist with the Suyker et al. (2004) report on rainfed and irrigated maize in eastern Nebraska, USA [8]. At the same growing stages the integration of canopy assimilated carbon and the accumulative carbon in dry biomass were higher in the MFR treatment than those in the CK treatment. This phenomenon can be also explained by that warmer and wetter soil in the MFR treatment stimulates assimilation more than respiration, which resulted in greater biomass accumulation of the MFR than that of the CK.

### Carbon Budget of Maize Ecosystem

In this region, at the maize harvesting stage there are often two harvest options for aboveground biomass while remaining all roots in the ground. Firstly, only grains are harvested while all of maize stalks were sliced and returned to the field. Thus only carbon in grains is eventually consumed and released back into the atmosphere. Secondly, all of aboveground biomass including grain are harvested and finally consumed as feedstuff for animals. So, all carbon in aboveground biomass is consumed and returned into the air. During the experiment of this study we used the first one, but we still calculated and compared carbon balance for maize ecosystem under these two practice options (Table 6) to evaluate the coupling effects of different harvest and tillage patterns. For the first option, the carbon sequestration of ecosystem switched from a strong sink (-0.644 kgC/m^2^ and -0.565 kgC/m^2^ for MFR and CK, respectively) to a weak sink (-0.208 kgC/m^2^ and -0.178 kgC/m^2^ for MFR and CK, respectively) for both treatments and three years. Net fixed carbon of ecosystem was greater by 16.9% at MFR than CK in the end. However, in the second option it reversed from a strong sink (-0.644 kgC/m^2^ and -0.565 kgC/m^2^ for MFR and CK, respectively) to a strong source (0.563kgC/m^2^ and 0.452 kgC/m^2^ for MFR and CK, respectively). Net released carbon of ecosystem from MFR also was higher by 24.6% than CK due to greater aboveground biomass. These results suggested that tillage as well as harvest patterns of aboveground biomass significantly changed the carbon balance of ecosystem in the end.

**Table 6:** Net carbon storage for spring maize during growing seasons of 2011–2013 after subtracting carbon in the harvested total aboveground biomass or grain with returning the sliced biomass to field.

Year	Treatments	Aboveground biomass (kg/m^2^)	Carbon in aboveground biomass (C_a_) (kg C /m^2^)	Grain yield (kg/m^2^)	Carbon in grain (C_gr_) (kg/m^2^)	NEE (kgC/ m^2^)	NEE+C_a_ (kg C/ m^2^)	NEE+C_gr_ (kg C /m^2^)
2011	CK	2.070	0.925	0.940	0.355	-0.532	0.393	-0.177
	MFR	2.380	1.064	1.070	0.404	-0.608	0.456	-0.204
2012	CK	2.420	1.082	1.081	0.408	-0.540	0.542	-0.132
	MFR	2.960	1.323	1.212	0.458	-0.616	0.707	-0.159
2013	CK	2.340	1.046	1.052	0.397	-0.625	0.421	-0.227
	MFR	2.760	1.234	1.176	0.444	-0.706	0.527	-0.262
Averages	CK	2.070	1.018	0.940	0.387	-0.565	0.452	-0.178
	MFR	2.380	1.207	1.070	0.435	-0.644	0.563	-0.208

Notes: CK and MFR stand for planting in non-mulching with flat tillage and partial plastic film mulching with furrow-ridge tillage, respectively. C_a_, f_w_, f_c_ and Y_gr_ stand for carbon in aboveground biomass, water content, carbon fraction and grain yield, respectively. C_gr_ = (1-f_w_/100) ×f_c_× Y_gr_, where f_w_ is 15.5%, f_c_ is 0.447 and Y_gr_ is grain yield; C_a_ = f_c_×Y_bio_, where f_c_ is 0.447 and Y_bio_ is biomass yield.

## Discussion

In cultivated cropland, improved tillage can enhance net ecosystem exchange of CO_2_ (NEE) and increase C sequestration in the soil. Thus, it is being regarded as the best promising solution to offset the rising of atmosphere by means of mitigation of greenhouse gas emission and sequestration of C in the cropland soil [1–3]. This study investigated the effects of newly and conventional tillage options through contrasting a partial plastic film mulching with furrow ridge tillage and non-mulching with flat tillage. It was supposed that the contrast in practices caused the different carbon exchange and balance between the improved and traditional tillage. In this study, to test precisely this hypothesis two set of eddy covariance were used to synchronously measure net ecosystem exchange of CK and MFR at one site.

### The Effects of MFR on Soil Water and Heat Conditions

Exclusive of the initial of experiment in 2011, MFR always had higher values of top soil water contents than CK (Fig 1). Moreover, there were some differences of top soil temperature between MFR and CK, which changed with maize growing stages. During the early growing stages (May through July) partial plastic film mulching mainly controlled resistance of heat flux between ecosystem and atmosphere, which resulted in that MFR had significantly higher soil temperature than in CK. However, due to coupling effects of canopy cover and partial plastic film mulching on this resistance MFR had similar values to CK in the middle growing stages (August) and slightly higher values than CK during the late growing stages (September). Totally, MFR increased the top soil temperature and water content by average values of 1.3°C and 4.3%, respectively, during the whole growing stages (Table 1), which is explained by that MFR ameliorated the processes of rainfall-infiltration-runoff and also increased the water and heat resistance at soil-air interface by the means of plastic film mulching, which resulted in conserving more water and heat in the soil [9]. These results are similar to several crops experiments on MFR such as maize [11, 25], cotton [7], watermelon [26], potato [27] and alfalfa [28]. However, the increments of soil water and temperature were different because of diversity soil, variable climate and crops varieties.

### Net Ecosystem Exchange of CO_2_ and Biomass Accumulations in MFR and Conventional Tillage

Compared to CK, the warmer and wetter soil advanced maize seedling and growth stages, and thus let GLAI seasonally matched better with temporal distribution of PAR in MFR. Therefore, more CO_2_ was assimilated in MFR than CK due to significantly correlation between NEE and GLAI, PAR, soil water content and temperature (Table 4). In MFR the canopy photosynthesis rate and ecosystem respiration were higher by 7.0% and 2.9%, respectively, which was estimated from eddy covariance measurements, than those in CK (Figs 4 and 5). The increments of integrated canopy photosynthesis were greater than those of integrated ecosystem respiration in MFR at different growing stages due to photosynthetic overcompensation mechanism and acclimatization of soil respiration under warming [29–30], causing a higher net ecosystem exchange of CO_2_ than CK.

Therefore, greater net ecosystem exchange of CO_2_ promoted higher biomass accumulation and larger green leaf area index (Figs 2 and 5). In the three whole growing seasons, MFR had greatly higher aboveground and total biomass, and slightly higher belowground biomass than CK. However, due to higher soil water status MFR had lower the percentage of biomass allocated to roots than CK. The difference of total biomass and its components between CK and MFR treatments changed with total amount and distribution of rainfall during three growing seasons. Li et al. (2012) also reported the similar results on biomass accumulation in cotton field under plastic mulching with drip irrigation in Xingjiang of northwest China [7]. Because of quicker growth and shorter growing season (Table 1) MFR had significantly greater GLAI than CK during the early and mid growing season but close to or higher in the late growing season. Certainly, larger green leaf area in MFR should intercept more PAR and assimilated greater CO_2_ than CK under the critical value of GLAI (≤3.0).

Canopy assimilated carbon significantly (p<0.001) correlated to that in biomass with linear relationship for both treatments and three years, which suggested that carbon fixed by ecosystem can be estimated from both eddy covariance measurements and biomass sampling. Theoretically, the slopes of linear relationships should be less than 1.0 due to plant respiration for both treatments. However, they were slighter greater than 1.0 for that eddy covariance system often underestimates net ecosystem exchange of CO_2_ [8].

### Carbon Budget in MFR and Its Comparison with Other Studies

With respect to the carbon released by harvested grain, the spring maize ecosystem acted as a carbon sink for both treatments and three years (Table 6). Moreover, while only considering the carbon in harvested grain MFR showed a higher net carbon sequestration by -30g C/m^2^ than CK. Our results is consistent with the reports of Hollinger et al. (2005) on no-tilled maize[31], Verma et al. (2005) on rainfed maize[32], Glenna et al. (2010) on reduced tilled maize[33], and Shen et al. (2013) on irrigated summer maize [34](Table 7). However, Baker and Griffis (2005) reported that maize ecosystem behaved as a weak sink and carbon source after subtracted the respiration of follow period at annual scale [5]. Compare with these results on maize fields, there are more or less differences of carbon budget of maize ecosystem at different sites, which can be explained partially by the geographical location, climate zone, soil type, cropping system, tillage, irrigation, data processes method and other factors vary different in experimental sites.

**Table 7:** Net ecosystem exchange, harvested grain carbon and carbon balance at different climate zone and for different tillage and maize varieties.

Location	Climate	Soil type	Management practices + Crop	Year	NEE (kgC /m^2^)	Carbon in grain (kg C /m^2^)	NEE+C_a_ (kg C /m^2^)	NEE+C_gr_ (kg C /m^2^)	Reference
Bondville, USA	humid continental climate-cool summer	fine-silt/ fine	no-till spring maize	1997, 1999, 2001 ^a^	-0.772	0.392		-0.380	Hollinger et al. (2005)
Mead, USA	humid continental climate-worm summer	silt clay loam	irrigated spring maize	2001–2004	-0.517~-0.381	0.470~0.521		-0.047~0.140	Verma et al (2005)
Mead, USA	humid continental climate-worm summer	silt clay loam	rainfed spring maize	2001,2003	-0.510,-0.397	0.335,0.297		-0.175,-0.100	Verma et al (2005)
Rosemount, USA	humid continental climate-cool summer	silt loam	reduced tillage spring maize	2003	-0.300	0.295		-0.005	Baker and Griffis (2005)
Rosemount, USA	humid continental climate-cool summer	silt loam	convention tillage spring maize	2003	-0.290	0.320		0.030	Baker and Griffis (2005)
Manitoba, Canada	humid continental climate-cool summer	clay	reduced tillage spring maize	2006	-0.720	0.512		-0.208	Glenna et al. (2010)
Lamasquere, France	humid subtropical climate	clay	irrigated silage maize	2006	-0.186±0.042	0.558±0.120	0.372 ±0.078		Béziat et al. (2009)
Luancheng, China	mid-temperate zone	silt loam	irrigated summer maize	2008–2011	-0.393~-0.338	0.226~0.343		-0.112~-0.050	Shen et al (2013)
Weishan, China	mid-temperate zone	silt loam	irrigated summer maize	2006, 08–09	-0.482~-0.360	0.255~0.323		-0.227~0.062	Shen et al (2013)
Shouyang, China	semiarid mid-temperate zone	clay loam	non mulching spring maize	2011–2013^a^	-0.565	0.387	0.452	-0.178	This study
Shouyang, China	semiarid mid-temperate zone	clay loam	plastic mulching spring maize	2011–2013^a^	-0.644	0.435	0.563	-0.208	This study

^a^. mean value in growing season.

After considering another option to harvest whole aboveground biomass as feedstuff, carbon balance of ecosystem will shift from a strong sink to a great source for both treatments and three years. Compared with CK, MFR changed from a slight stronger sink (-30gC/m^2^) to a greater source (111 gC/m^2^). This reversal change of carbon balance suggested that the options for aboveground biomass harvest and tillage complicatedly affect the uptake and release of carbon dioxides in cropland. It is similar to the result on silage maize (372±78 gC/m^2^) reported by Béziat et al. (2009)[35]. Therefore, it should be required to combine partial plastic film mulched furrow-ridge tillage with returning the stalk to the field at harvest in the similar regions to this study site. Thus, this practice can fix more carbon in the soil, and alleviate the rise of atmosphere carbon dioxide while boosting the grain yield.

## Conclusions

This study has qualified the effects of two management practices, non-mulching with flat tillage and partial plastic film mulching with furrow-ridge tillage, on the soil water and temperature, NEE, biomass accumulation and ecosystem carbon balance of rain-fed maize, the dominant single cropping system, on the semiarid region of China Loess Plateau. Partial plastic film mulching with furrow-ridge tillage enhanced significantly soil water and temperature, especially at the early growing season, which promoted the crop development and advanced the whole growing season. Eddy covariance measurements indicated that MFR can stimulate assimilation more than respiration at ecosystem scale during whole growing season, resulting in higher carbon storage in terms of NEE. With respect to carbon in harvested grain (or aboveground biomass), there is a slight higher carbon sink (or a stronger carbon source) in MFR than CK. A more effective approach may be partial plastic film mulching with furrow-ridge tillage while returning the stalks of maize to the soil at harvest, which allocates more of biomass carbon to soil carbon storage.
